# Predatory publishing in radiology: advice for early-career researchers

**DOI:** 10.1186/s13244-026-02435-w

**Published:** 2026-11-09

**Authors:** Uliana Pidvalna, Daniel Pinto dos Santos

**Affiliations:** 1 Ukrainian-Polish Heart Center Lviv, Lviv, Ukraine; 2 Danylo Halytsky Lviv National Medical University, Lviv, Ukraine; 3 University Medical Center of the Johannes Gutenberg University Mainz, Mainz, Germany

**Keywords:** Predatory journals as topic, Ethics, Pseudoscience, Publishing, Radiology

## Abstract

**Abstract:**

Predatory journals typically use practices based on deceit, misleading information, lack of transparency, and the absence of rigorous peer review. Within radiology, such journals frequently publish articles with minimal editorial oversight and limited scientific credibility. The relevance of this issue in radiology has grown as imaging research has expanded rapidly, particularly in artificial intelligence (AI), quantitative imaging, and subspecialty-driven innovation. Given the academic pressure created by the “publish or perish” culture, this environment makes early-career radiologists especially vulnerable to predatory journals that promise rapid publication and high acceptance rates. Predatory journals exploit this environment by sending unsolicited invitations to publish or join editorial boards, frequently using flattering language and promising unrealistic publication timelines. These journals generally lack robust peer review, methodological scrutiny, and transparency and are typically not indexed in established databases such as PubMed, Scopus, or Web of Science. Consequently, articles published in these outlets have reduced visibility, limited citation potential, and minimal impact on radiological science or clinical practice. Furthermore, publication in predatory journals may adversely affect academic reputation and career advancement, as hiring and funding committees increasingly prioritise journal quality and publication integrity. The rapid expansion of radiology research, particularly in diagnostic imaging, interventional radiology, and AI-powered applications, may increase opportunities for the dissemination of low-quality or insufficiently validated evidence through predatory journals. Raising awareness of predatory publishing practices and consistently applying evaluation tools such as the “Think. Check. Submit.” framework, are fundamental to safeguarding scientific integrity and ensuring the dissemination of high-quality radiological research.

**Key Points:**

***Question*** Predatory publishing is a growing concern in radiology, driven by rising publication pressure and the “publish or perish” culture. Early-career researchers are particularly susceptible to unethical journals that offer rapid publication.

***Findings*** Predatory journals frequently bypass rigorous peer review, lack transparency, and are often not indexed in major databases. These journals exploit authors through unsolicited invitations and prioritise profit over scientific quality.

***Critical relevance statement*** Avoiding predatory journals is important for protecting research credibility, career progression, and scientific integrity. Publications in these outlets may disseminate unreliable evidence and are often disregarded in academic and clinical evaluations.

**Graphical Abstract:**

**Figure:**
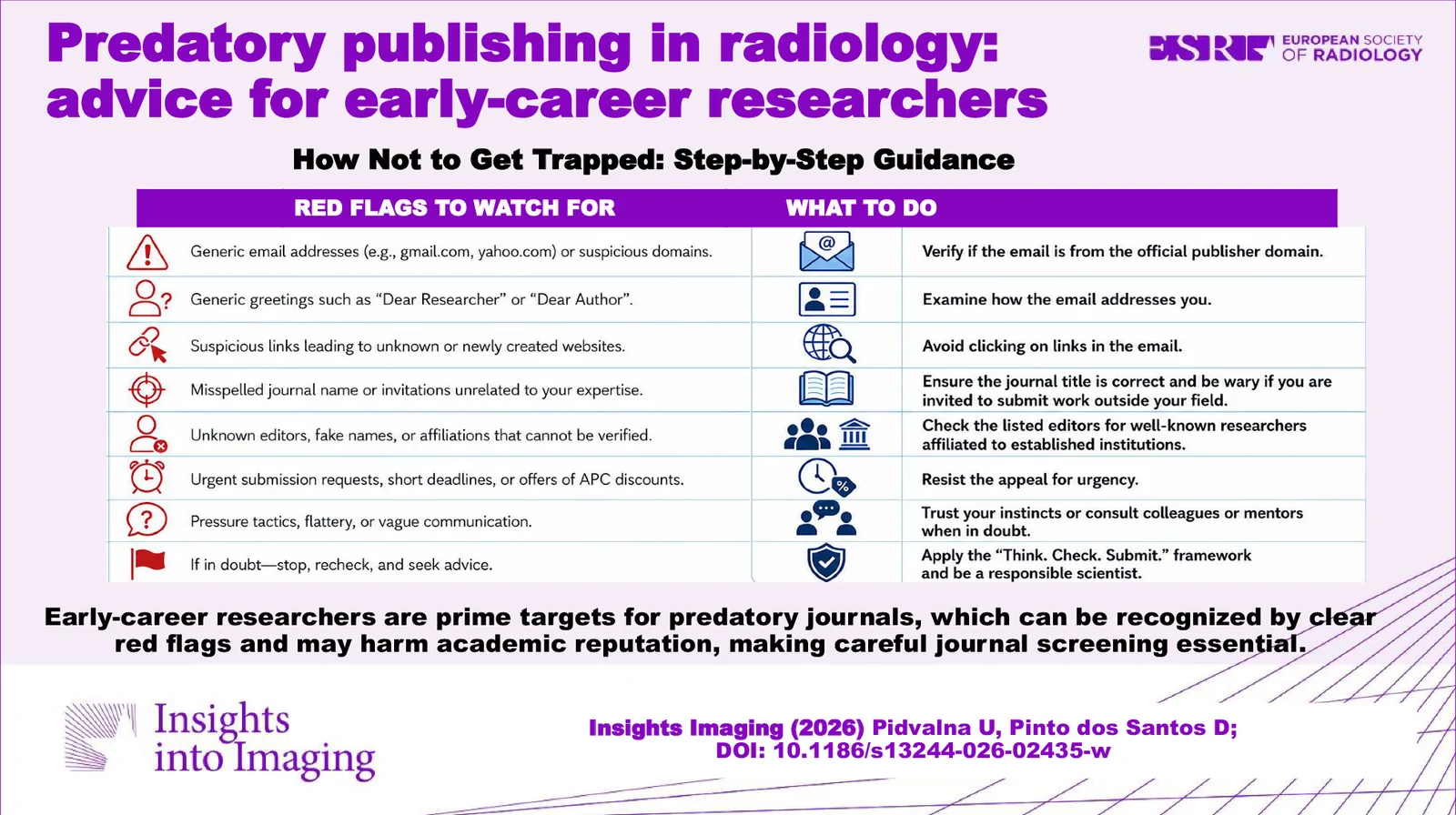

## Introduction

Predatory publishing is characterised by the use of “…false or misleading information, deviation from best editorial and publication practices, a lack of transparency…”[1]. According to Medical Subject Headings (MeSH) Description Data 2025, “predatory journals as topic” refers to “Works about medical journals that publish articles online with little or no peer review, low academic standards, and little credibility”[2].

As radiology research becomes more competitive, early-career investigators often feel pressured to publish quickly and frequently to advance their professional careers—a phenomenon typically referred to as “publish or perish” culture [3, 4]. Some publishers capitalise on this pressure by promising rapid turnaround times or high acceptance rates, practices that may indicate predatory publishing [5]. Modern medical science should always be grounded in evidence and have high academic standards [6]. Therefore, early-career researchers should avoid predatory publications, as they can have lasting detrimental effects on their reputations [7].

After publishing an initial manuscript, authors may receive numerous email invitations to submit their work to various journals or join editorial boards [5, 8]. It is common practice to collect contact information from publicly available sources, such as articles and conference submissions, to directly advertise to authors [9]. While some invitations are legitimate, predatory publishers prioritise profit over scientific integrity [10]. Unfortunately, the number of potential or probable predatory journals in radiology is increasing rapidly [11]. The line between legitimate and predatory journals is not always easy to draw [12]. Unlike reputable journals, which rely on rigorous peer review [13, 14] and established academic networks, predatory publishers exploit publicly available information and craft flattering invitations to create the illusion of recognition. Awareness of these tactics is essential for maintaining academic integrity and avoiding illegitimate journals.

## Why is it important to avoid predatory journals?

Publishing in predatory journals can undermine a researcher’s academic integrity and may be disregarded in grant applications and fellowship reviews, limiting career advancement [7]. Furthermore, predatory journals are typically not indexed in reputable databases such as PubMed, Scopus, or Web of Science, reducing the likelihood that the research published there will be discovered, cited, influence clinical practice, or guide future studies. Even worse, researchers can unintentionally support unethical practices of poor peer review and ultimately publication of misinformation. This is compounded by the fact that many predatory journals charge high publication fees.

## Radiology-specific vulnerability to predatory publishing

Bibliometric analyses reveal a consistent rise in suspected predatory journal publications across biomedical fields, including radiology [4, 8, 15].

Radiology is especially susceptible to predatory publishing given the rapid surge of imaging research, particularly in artificial intelligence (AI) and quantitative imaging [15, 16]. In this competitive landscape, the pressure to publish in high-impact journals and the rapid expansion of AI research can shift the focus from methodological rigour to rapid dissemination. Predatory journals exploit this by offering expedited publication for imaging and AI studies, and they often lack adequate peer review, methodological transparency, or substantive validation (Table 1).

**Table 1:** Key differences between legitimate and predatory journals in radiology publishing

Feature	Legitimate journals	Predatory journals
Peer review	Rigorous, transparent	Minimal or absent
Editorial board	Recognized experts	Fake or unverifiable
Indexing	PubMed, Scopus, Web of Science	Misleading or false claims
Article processing charges transparency	Clearly stated	Hidden or inconsistent
Review time	Weeks to months	Days to a few weeks
Scope	Clearly defined	Broad, inconsistent
Email solicitation	Rare	Aggressive spam invitations

Radiology subspecialties each face unique vulnerabilities. In interventional radiology, case reports with minimal methodological scrutiny may be readily accepted by predatory journals. In diagnostic neuroradiology, image-rich studies are targeted for their visual impact and perceived novelty. In the emerging field of AI-focused radiology, predatory journals frequently publish deep learning studies without requiring external validation, adherence to reproducibility standards, or comprehensive methodological reporting.

## Red flags for predatory journals

Most importantly, predatory publishers often use non-institutional email accounts (e.g., Gmail, Outlook) or obscure domains. Messages may be poorly or oddly formatted and lack personalisation (e.g., not mentioning the target author’s name). It is not uncommon to receive invitations to publish in journals outside the target author’s expertise. To make the invitation attractive, promises of “fast” or “friendly” review processes are made, often implying minimal to no scientific evaluation. This is further underscored by unrealistic, urgent timelines or special discounts on article processing charges (APCs), leading readers to believe they might otherwise miss out on this opportunity. Short submission deadlines or guaranteed acceptance within days or even hours should be an immediate warning sign, as honest and detailed peer review requires substantial amounts of time (often months).

Invitation emails typically include links to journal websites, supposed editorial board members, and indexing claims to give the invitation a more legitimate appearance. However, closer inspection can reveal additional warning signs, such as broken links, grammatical mistakes, inconsistent formatting, and unclear instructions for authors. Predatory publishers might also falsely assert indexing in PubMed, Scopus, or Web of Science. Verifying the actual indexing status directly on the respective platforms can serve as an additional check.

Similarly, researchers should exercise caution when receiving invitations to join editorial boards. Although such appointments may appear beneficial, predatory publishers may use them to enhance the journal’s perceived credibility, potentially exposing researchers to reputational risks [9].

## How not to get trapped: step-by-step guidance

Although a proposal may seem appealing and promising, researchers should carefully assess any unsolicited invitation from a journal or publisher. Paying close attention to these points can help safeguard academic integrity and avoid predatory schemes.

- Verify if the email is from the official publisher domain [1, 14].
- Examine how the email addresses you. Genuine invitations usually include your full name and title.
- Avoid clicking on links in the email. Search for the journal directly in reputable databases [11].
- Ensure the journal title is correct and be wary if you are invited to submit work outside your field [9].
- Check the listed editors for well-known researchers affiliated with established institutions.
- Resist the appeal for urgency. Short deadlines and APC discounts are rarely offered by legitimate journals [11, 15].
- Trust your instincts or consult colleagues or mentors when in doubt.
- Apply the “Think. Check. Submit.” framework [17] and be a responsible scientist [18].

Conclusions. Predatory publishing presents major risks to early-career radiology researchers. Awareness and careful journal selection by checking indexing services, consulting trusted directories, evaluating editorial boards, and applying the “Think. Check. Submit” framework help ensure research is published in reputable journals. Establishing solid publishing practices early is crucial for career growth and preserving the integrity of scientific communication in radiology.
